# Current Approaches to Alkyl Levulinates *via* Efficient Valorization of Biomass Derivatives

**DOI:** 10.3389/fchem.2020.00794

**Published:** 2020-10-15

**Authors:** Xiaofang Liu, Wenjia Yang, Qiuyun Zhang, Can Li, Hongguo Wu

**Affiliations:** ^1^Guizhou Provincial Key Laboratory for Rare Animal and Economic Insects of the Mountainous Region, College of Biology and Environmental Engineering, Guiyang University, Guiyang, China; ^2^School of Chemistry and Chemical Engineering, Anshun University, Anshun, China; ^3^State-Local Joint Laboratory for Comprehensive Utilization of Biomass, Guizhou University, Guiyang, China

**Keywords:** alkyl levulinates, levulinic acid, furfuryl alcohol, chloromethyl furfural, acidic catalysts

## Abstract

Biomass is a potential non-food, carbon-neutral, and abundant resource, which can be used as an alternative to fossil fuels during the sustainable preparation of various platform chemicals. Alkyl levulinates (ALs) have found widespread application as flavorings, plasticizing agents, and fuel additives, as well as synthetic precursors to various building blocks. Several processes have been investigated to transform biomass and its derivatives into ALs, which mainly include: (i) direct esterification of levulinic acid (LA) with alkyl alcohols and (ii) alcoholysis reactions of renewable biomass feedstocks and their derivatives, including furfuryl alcohol (FAL), chloromethyl furfural (CMF), and saccharides. This review focuses on illustrating the effects of the biomass pretreatment step, catalyst texture, possible mechanisms, acidities, and intermediates on the synthesis of ALs from sustainable resources covering a wide range of intermediates, including diethyl ether (DEE), 4,5,5-triethoxypentan-2-one (TEP), ethoxymethylfuran (EMF), ethyl-D-fructofuranoside (EDFF), and ethyl-D-glucopyranoside (EDGP).

## Introduction

As the natural reserves of non-renewable resources such as petroleum, diesel, natural gases, and coal (fossil fuels) dwindle (Badgujar and Bhanage, 2015; Dhyani and Bhaskar, 2018) while causing unavoidable environmental issues, such as the emission of harmful gases and global-warming (Sun and Cheng, 2002; Wagh et al., 2016), extensive efforts must be devoted toward the search for alternative and renewable resources that are also environmentally friendly. Biomass is a carbon source used for renewable energy, which can provide multiple fuels, chemicals, and value-added platform molecules in a green and sustainable manner (Rackemann and Doherty, 2011; Tadele et al., 2017; Badgujar and Bhanage, 2018). Alkyl levulinates (ALs) derived from biomass have shown great potential for biorenewable fuels like bio-lubricants (Mukherjee et al., 2015), chemicals synthesis (Mullen et al., 2013), polymer or resin precursors (Alloaoua et al., 2014; Cousinet, 2014), green solvents (Lomba et al., 2011), plasticizers (Bloom, 2007), food-flavor agents (Yontz, 2011), and pharmaceuticals (Tsucha and Yoshida, 1994) during the effective utilization of biomass (Table 1). (Fiorentino et al., 2014). An investigation on Scopus indicated that the interest in developing ALs as a fuel has built up great momentum over the past 5 years. Research on ALs has mainly concentrated on methyl, ethyl, and butyl levulinate (Mascal and Nikitin, 2010b). Among these three levulinates, ethyl levulinate (EL) exhibits enhanced solubility with diesel (Christensen et al., 2011) and only ~4% NO_x_ emissions upon blending in diesel (Windom et al., 2011). Therefore, research has mostly been devoted toward the effective synthesis of EL.

**Table 1:** Potential alkyl levulinates applications.

**Entry**	**Applications**	**Products**
1	Chemical industry	Chiral reagent, polyhydroxy alkanoates, lubricants, adsorbents, formic acid, valerates
2	Fuels and fuel additives	EL, 2-methyltetraydrofuran, γ-valerolactone, angelica lactone, methyl levulinate, and other esters
3	Pharmaceuticals	δ-aminolevulinic acid, calcium levulinate, heterocylic derivatives of levulinic acid, angelica lactone, ketals, tetrapyrroles, succinic acid
4	Food additives	γ-valerolactone, ethyl valerate, succinic acid, valerate esters
5	Agricultural products	δ-aminolevulinic acid, formic acid, lignins, ethyl formate
6	Solvents and polymers	Diphenolic acid, succinic acid, pyridine, furans, epoxies, 1,4-butanediol, tetrahydrofuran, N-methyl-2-pyrrolidone, -γ-butyrolactone

**Figure 1:**
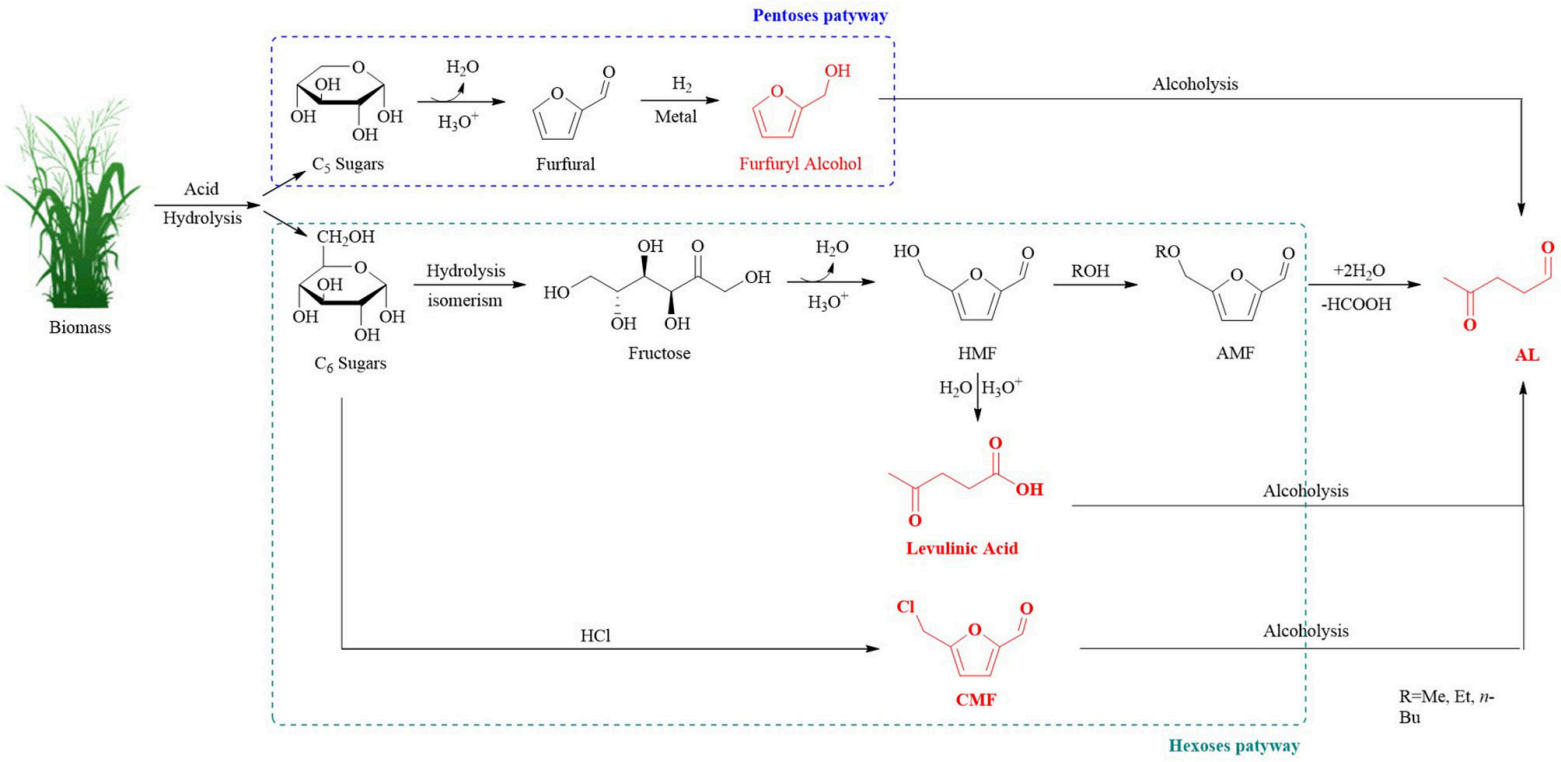
The pathways used for the production of ALs from bio-renewable resources.

ALs can be produced from biomass or via the conversion of levulinic acid (LA), furfuryl alcohol (FAL) (Démolis et al., 2014; Desidery et al., 2018), or chloromethyl furfural (CMF) (Mascal and Nikitin, 2010a). All of the possible reaction pathways involved in the alcoholysis procedure performed in the presence of acid catalysts have been reported (Figure 1). This review comprehensively contains all of the discussed approaches used for the production of ALs and executes critical evaluation of the various types of catalysts used in the conversion reactions: i) an overall understanding of the reaction parameters, texture and chemical properties, possible mechanism, and the reaction intermediates that favor the development to achieve high yields (Chia et al., 2013; Gupta et al., 2016), ii) different catalytic routes of ALs preparation, iii) utilization of various catalysts to obtain ALs, and iv) the future challenges and opportunities for the lab scale to the industry scale development of ALs.

## Preparation of ALs

Numerous advantages for using solid acid catalysts have been demonstrated including their low cost, ease of recycling from the product mixture, low equipment corrosion, and high thermal stability (Su et al., 2013b), while the application of heterogeneous materials toward the production of ALs requires further detailed discussion including the reaction temperature, time, substrates, acidic density and intensity, and textural properties.

### Routes to Prepare ALs From LA

#### Heteropolyacid (HPA) as Catalysts

The LA esterification reaction has been investigated using several solid acid catalysts toward the production of ALs in comparatively high yields (Pileidis and Titirici, 2016). Heteropolyacid (HPA) is a special species of acid composed of a combination of certain metals (tungsten, molybdenum, or vanadium) and *p*-block non-metals (silicon, phosphorus, or arsenic) with acidic hydrogen and oxygen atoms, which are generally applied as reusable acid catalysts for the preparation of fine chemicals (Yan et al., 2013; Wu et al., 2016a,b,c; Zhou et al., 2016; Manikandan and Cheralathan, 2017; Ramli et al., 2017a; Zheng et al., 2017; Luan et al., 2018; Vilanculo et al., 2018; Lucas et al., 2019). Numerous structural combinations can be prepared upon adjusting the metal and non-metal used. Furthermore, HPAs possess very strong Brønsted acidity and can be applied as homogenous or heterogeneous catalysts and solvents (Wu et al., 2016b; Zhou et al., 2016; Ramli et al., 2017a; Zheng et al., 2017).

HPAs have several disadvantages, including high solubility in water and other polar solvents, low specific surface area and thermal stability, and difficult reusability and regeneration (Gupta and Paul, 2014; Hu et al., 2015; Yamaguchi and Shirai, 2016), which hinders their application as efficient and effective catalysts for reactions with large molecules. A variety of diverse synthetic approaches have been studied to overcome these disadvantages including their immobilization on ordered silica, zirconia, and niobium (Narkhede et al., 2015).

HPA implanted into the Wells–Dawson (WD) structure provided EL with a 76% yield upon heating at 78°C for 10 h (Pasquale et al., 2012). Luan et al. carried out the production of ethyl, methyl, and isobutyl levulinates using an organic-salt of H_4_SiW_12_O_40_ as the catalyst (Luan et al., 2018). Manikandan and Cheralathan investigated heteropoly acid-supported silicalites in the synthesis of various ALs (Manikandan and Cheralathan, 2017).

Similarly, when changing WD to Keggin HPA (H_3_PMo_12_O_40_), the EL yield increased to 93% under identical reaction conditions. The higher yield observed over Keggin HPA (H_3_PMo_12_O_40_) was attributed to its higher acidity when compared to the WD structure. While transforming the alkylation substrates with isobutylene and methanol, WD HPA showed increased activity during the preparation of ALs when compared to Keggin HPAs, which was attributed to the higher adsorption of the reactants in the WD structure (Briand et al., 2003). Nevertheless, both WD- and Keggin-structured HPAs show comparable or higher activity than their corresponding homogeneous catalysts (Baronetti et al., 1998). However, leaching of the HPAs in polar solvents still occurs owing to the weak interactions formed between the HPAs and the support. The Keggin HPA (H_4_SiW_12_O_40_) embedded in the channels of mesoporous SiO_2_ gave EL a 67% yield with improved recyclability when compared to the original HPA (Yan et al., 2013). Similarly, a combination of HPAs and zeolites was reported by Nandiwale et al. Improved stability was achieved using dodecatungstophosphoric acid (DTPA) supported on desilicated HZSM-5, which gave EL a 94% yield from LA at 76°C in 240 min (Nandiwale et al., 2013). High EL yields up to 77% were achieved over four reaction cycles demonstrating the high stability of the catalyst.

Neurock, Iglesia, and co-workers carried out a series of investigations on the acidity and reactivity of Keggin HPAs, which confirmed that the deprotonation energy (DPE) of the HPA was crucial for its activity (Macht et al., 2007, 2008). The HPA DPE determines the strong acidity of the material and thereby its increased reactivity in acid-catalyzed processes. The DPE of Keggin H_4_SiW_12_O_40_ was determined to be 1,105 kJ·mol^−1^, which was lower than Keggin H_3_PMo_12_O_40_ (1,126 kJ·mol^−1^) (López et al., 2012). Hence, the two Keggin-structured HPAs prove the correlation between their activity and DPEs. To further enhance the acidity of Keggin HPAs, an acidic silica-like support such as ZrO_2_ was applied for the preparation of a variety of hybrid Keggin HPA/ZrO_2_ composites containing both Brønsted and Lewis acid sites with increased activity toward the production of ALs when compared to HPA-silica (Su et al., 2013a). DRIFTS research on H_3_PW_12_O_40_/ZrO_2_ further verified the existence of Brønsted and Lewis acid sites, which was in accordance with this hypothesis (Alsalme et al., 2010; Wu et al., 2016a). In addition, the modification of Keggin HPA- organosilica/ZrO_2_ was proposed to prepare a hybrid catalyst, whereby a maximum AL yield of up to 95% was observed (Luan et al., 2018). Subsequently, several efforts have been devoted toward designing catalysts for the transformation of LA into ALs with enhanced stability, available active sites, and recyclability.

#### Zeolites as Catalysts

**Figure 2:**
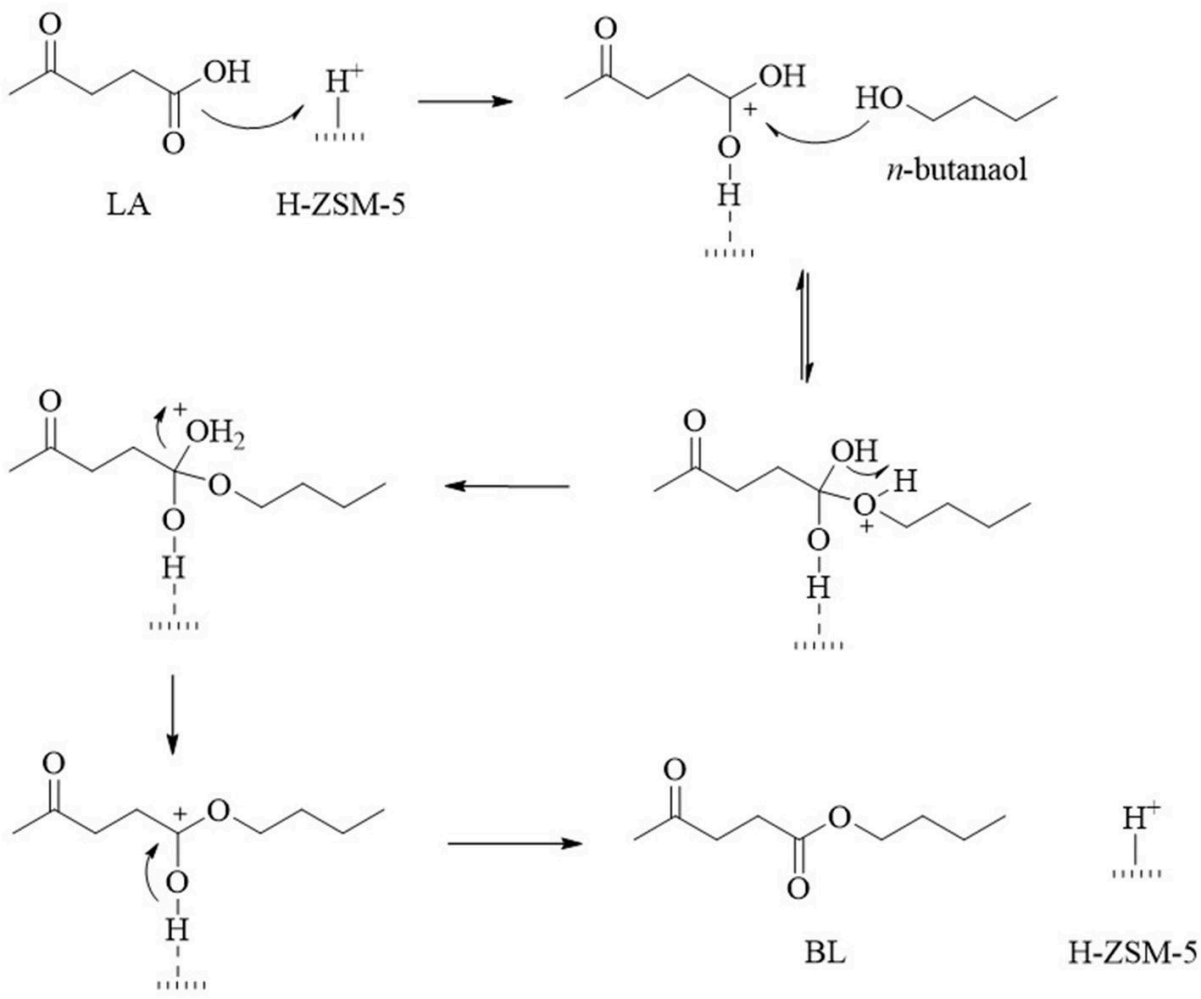
Proposed reaction mechanism for the esterification of LA with *n*-butanol over the H-ZSM-5 catalyst.

Other factors in regard to the catalyst that influence the reactivity and selectivity during the conversion of LA into EL are its texture, porosity, specific surface area, and availability of active sites. Well-organized materials, such as zeolites, contain functionalities that can be used to control the acidity and pore size to achieve better EL yields. For example, desilicated H-ZSM-5 (DH-ZDM-5) has moderate acidity (0.73 mmol·g^−1^), a high surface area (427.6 m^2^·g^−1^), and mesoporosity, which affords EL a 95% yield under autogenous pressure (Nandiwale et al., 2014). The obtained EL yield was comparable to that obtained over HPA/H-ZSM-5 at high temperature (130°C). To account for this phenomena, Janik et al. carried out DFT calculations on the mobility of the isolated protons. The results indicate the relatively low activation barrier of phosphotungstic acid (Ea = 103.3 kJ·mol^−1^) (Janik et al., 2005), which was also calculated by Ryder et al. for H-ZSM-5 (Ea = 117.2 kJ·mol^−1^) (Ryder et al., 2000). The activation barrier was confirmed to be significantly reduced (Ea = 11.2 kJ·mol^−1^) in the presence of hydrated protons in the Keggin HPA structures (Janik et al., 2005). The acid strength was directly proportional to the protons mobility and thus, a slightly higher reaction temperature was required for the conversion of LA into EL using desilicated H-ZSM-5 (Nandiwale et al., 2014). Dharane and Bokade reported the production of butyl levulinate using dodecatungsten phosphoric acid inserted in acid-treated clay (K-10) (Dharne and Bokade, 2011). Therefore, the lower proton mobility and pore structure of zeolites lead to the higher selectivity observed toward EL during the esterification of LA, which was attributed to the highly efficient mass transfer observed within the moderate pore channels in zeolites (Yan et al., 2013). Patil et al. further demonstrated that mesoporous zeolites (micro/meso-HZ-5) with larger cavities enhanced the substrates access to the acidic sites (total acidity = 0.73 mmol·g^−1^) and gave a high EL yield of up to 95% when compared with a traditional microporous zeolite (H-BEA_0.10_; total acidity = 0.69 mmol·g^−1^, EL yield = 39.2%) (Patil et al., 2014). The achievement was confirmed further by Nandiwale et al. on the esterification of LA with *n*-butanol (Nandiwale and Bokade, 2015), and the proposed mechanism is shown in Figure 2.

#### Metal Oxides as Catalysts

Mesoporous metal oxides are potential alternatives to zeolites as catalytic supports (Dave and Pant, 2011; Mondal et al., 2015). During the esterification for LA to prepare ALs, acidic catalysts are desirable. For instance, sulfated zirconia (SO4^2−^/ZrO_2_) exerts a higher Hammett acid strength (*H*_0_ = −16.04) than homogeneous pure sulfuric acid (*H*_0_ = −11.99), which indicates it is a superacid (Yadav and Nair, 1999). Owing to their high acidity, solid superacids embedded into various metal oxides have been developed toward the transformation of LA into ALs. Sulfated tin oxide gave EL a 44% yield, which was significantly better than that observed using non-sulfated tin oxide (6% EL yield) under identical reaction conditions (Fernandes et al., 2012). For the resulting sulfated oxides, the acidity followed the order of ${\text{SO}}_{4}^{2-}$/ZrO_2_ > ${\text{SO}}_{4}^{2-}$/Nb_2_O_5_ > ${\text{SO}}_{4}^{2-}$/TiO_2_ > ${\text{SO}}_{4}^{2-}$/SnO_2_. (Rao et al., 2006), while the EL yield trend observed with the sulfated metal oxides followed the order of ${\text{SO}}_{4}^{2-}$/SnO_2_ > ${\text{SO}}_{4}^{2-}$/TiO_2_ >${\text{SO}}_{4}^{2-}$/ZrO_2_ > ${\text{SO}}_{4}^{2-}$/Nb_2_O_5_ (Yadav and Nair, 1999). The acidic strength was opposite to the EL yield and could be attributed to the texture, specific surface area, and pore size of the catalyst, which in turn determines the accessibility of the active sites. Fernandes et al. carried out these investigations and the results suggested that the poor activity (14% EL yield) observed for the ${\text{SO}}_{4}^{2-}$/Nb_2_O_5_ catalyst can be attributed to its lower S_BET_ (67 m^2^·g^−1^) when compared to ${\text{SO}}_{4}^{2-}$/TiO_2_ (107 m^2^·g^−1^) and ${\text{SO}}_{4}^{2-}$/SnO_2_ (130 m^2^·g^−1^) (Fernandes et al., 2012). Similarly, the low S_BET_ (51.7 m^2^·g^−1^) of sulfated zirconia (${\text{SO}}_{4}^{2-}$/ZrO_2_) displays an EL yield of 27.5% at 70°C, which was significantly increased up to 80% when using mingling mesoporous silica (S_BET_ = 130 m^2^·g^−1^) synthesized via a successive co-precipitation-impregnation method. Optimization studies confirmed that ZrO_2_ with a Si content of 5.0–10 mol% Si per Zr exhibits the best catalytic performance (Kuwahara et al., 2014). However, when catalyzed using sulfated metal oxides such as ${\text{SO}}_{4}^{2-}$/ZrO_2_, ${\text{SO}}_{4}^{2-}$/SnO_2_, and ${\text{SO}}_{4}^{2-}$/Nb_2_O_5_ at elevated temperature (160°C), each catalyst showed improved EL yields (~70%), which was attributed to their enhanced activity (Yadav and Yadav, 2014). In addition, the higher density and availability of the active acid sites in the catalyst may enhance the EL yield (Li Z. et al., 2014). Dispersing TiO_2_ onto sulfated zirconia nanoparticles increases the sulfur complex density up to 1.71 sulfate groups per nm^3^ and when distributed on the surface of a ZrO_2_/TiO_2_ support results in a significant improvement when compared to their corresponding TiO_2_ nanocomposites (0.35 sulfate groups per nm^3^) (Li et al., 2012). Based on the above discussion, Kuwahara and co-workers reported a high EL yield of up to 80% from LA upon heating at 70°C over 1,440 min using sulfated Zr-SBA-15, which was attributed to the appropriate acid site density of sulfated ZrO_2_ immobilized on the ordered and mesoporous silica template (Kuwahara et al., 2013). Therefore, the acid site density, acid strength, and predominant morphological characteristics contribute to the higher EL yield. However, the drawback of deactivation via hydration of the sulfur complexes in sulfated metal oxide catalysts occurs in an aqueous phase or when water is produced as a product of the reaction, which impedes the development of numerous reactions.

#### Sulfonic Acid-Functionalized Materials as Catalysts

To overcome the limitations of sulfated metal oxides, sulfonic acid-based materials have been introduced as an alternative to performing the desired reactions. Sulfonic acid ions construct a hydrophobic microenvironment in the reaction medium, which protects the catalyst from water molecules generated during the reaction, thus guaranteeing their stability (Song et al., 2015). Therefore, sulfonic acid functionalized mesoporous materials have several advantages including high surface area, accessible active sites, appropriate pore structures, and high stability for achieving the desired ALs in good yield.

Silica has aroused great interest from researchers owing to its superior features, such as ordered porosity, low cost, adjustable surface functionalities, high surface area, and chemical and thermal stability (Melero et al., 2013; Maggi et al., 2016; Chermahini and Nazeri, 2017; Enumula et al., 2017; Ramli et al., 2018; Yang and Tang, 2019).

EL was obtained in 100% yield upon heating at 117°C for 2 h using propylsulfonic acid-functionalized mesoporous silica (Pr-SO_3_H-SBA-15), which could be reused three times without needing to be regenerated (Melero et al., 2013). Similarly, Ramli et al. prepared methyl levulinate in 69% yield over sulfated silica under mild reaction conditions (Ramli et al., 2018). Meanwhile, Chermahini and Nazeri reported the use of aluminum-containing MCM-41 for the production of isobutyl and butyl levulinate, and verified that the regenerated catalyst can be recycled without any obvious loss of activity (Chermahini and Nazeri, 2017). Besides, the insertion of tungsten oxide into SBA-16 also showed enhanced acidity and catalytic performance, and gave a higher yield of the desired levulinate products. This was attributed to the uniform distribution of acidic tungsten oxide sites over the large specific surface area and ordered structure of SBA-16 (Enumula et al., 2017). These materials combined with silica-based materials are easily deactivated in polar solvents because of the H-bonding formed between the active silica-functionalized groups and polar solvent molecules.

Inspired by these outstanding results, Oliveira et al. conducted studies using carbon nanotubes (CNT) as a support with a high surface area available for sulfonation. By varying the functionalization temperature and number of acid sites, the activity of the sulfonated CNT catalyst can be easily adjusted and controlled by its acid site density. Unfortunately, the overall EL yield was relatively low (~50%) because of the selectivity and strong chemisorption of LA on the CNT surface (Oliveira and Teixeira Da Silva, 2014).

An innovative approach to prepare bifunctional acid-base catalysts using Zr-containing metal-organic frameworks (MOFs) formed using 2-aminoterephthalate ligands has been proposed by Corma and coworkers (Cirujano et al., 2015). The interactions formed between the catalyst, LA, and alcohol were activated on the catalytic centers of the metal (Zr) and amino group in the ligand. The results demonstrate that these centers play different roles within proximity to one another and they all allowed the simultaneous activation processes to occur. The resulting NH_2_-Zr-MOF catalysts afford a high EL yield (>95%) under relatively mild reaction conditions (78°C). The excellent performance was further confirmed by the turnover frequency (TOF), which was measured to be 230 h^−1^, which was >2-fold higher than that obtained using homogeneous *p*-toluene sulfonic acid (TOF = 120 h^−1^).

Several research groups have explored the effectiveness of various heterogeneous catalysts toward the production of EL from LA under optimal reaction conditions. Sulfonated carbon nanotubes have also been explored as a catalyst for the synthesis of ALs (Oliveira and Teixeira Da Silva, 2014). However, this type of catalyst is not recyclable when employed in the LA esterification reaction and exhibits a lower catalytic performance than that observed using the classic benchmark catalyst (Amberlyst-15).

Resins are organo-polymeric materials, which possess high surface areas, high ion-exchange capacities, and various functionalities depending on the type of resin used (Tejero et al., 2016; Marrocchi and Vaccaro, 2017; Ramli et al., 2017b; Kokare et al., 2018; Trombettoni et al., 2018). The main catalytic resins are Amberlyst-15, Amberlyst-16, Amberlyst-36, Amberlyst-70, Purolite, Dowex, and polystyrene-supported *p*-toluensulfonic acid. The existence of sulfonic-acid groups (-SO_3_H) endows the resin catalysts with acidic properties, which initiate the conversion process (Tejero et al., 2016; Ramli et al., 2017b; Kokare et al., 2018). Ramli et al. developed an ion-exchange resin (Amberlyst-15) for use as a solid acid catalyst in the production of ALs under safe media flow conditions, confirming that the process was chemically and environmentally efficient (Ramli et al., 2017b). Kokare et al. carried out response surface optimization for the preparation of *n*-butyl levulinate over Amberlyst-15 and a maximum LA conversion of 97% was achieved using a LA to *n*-butanol molar ratio of 1:4 at 124°C (Kokare et al., 2018). Meanwhile, a low divinylbenzene (DVB) content gel-type resin (Dowex 50Wx2; 2% DVB) gave good yields of the desired AL products, which was attributed to the accessibility of the reactants to the acid centers in the highly swollen and low polymer density resin (Tejero et al., 2016). The reusability of Amberlyst-15 and Amberlyst-70 is highly practical and requires a simple washing step, and are therefore less expensive when compared to other resin-based catalysts. However, the instability of Amberlyst-15 at high temperatures greatly impedes its further application. The major challenges for the employment of resins in these catalytic conversion reactions are their high cost, thermal instability, non-flexibility, H-bond formation, and destabilization of the active centers.

### Routes to Prepare ALs From Biomass Feedstocks and Their Derivatives

#### FAL as a Feedstock

Zhang et al. have explored a wide variety of acid catalysts including zeolite, ion-exchange resins, ionic liquids (ILs), and HPA toward the synthesis of butyl levulinate (BL) (Zhang et al., 2011). The results showed that the production of 2-butoxymethylfuran (BMF) was faster than the subsequent conversion reaction to form BL. In addition, reports have speculated that BMF reacts with water, which accelerates the reaction. When one equivalent of water was added to the reaction, BL was obtained in 93% from FAL. This confirmed the higher activity observed in the presence of water when compared to the reaction performed in the absence of water (BL yield = 88%). Based on their experimental results, a proposed mechanism for the conversion of FAL into ALs indicated that the reaction may occur via the formation of an alkoxymethylfuran intermediate.

Similar conclusions have been reported by Huang et al. when studying the conversion of FAL into methyl levulinate (ML) (Huang et al., 2016). The results indicated multiple routes involving the production of methoxymethylfuran (MMF) and 4,5,5-trimethoxypentan-2-one (TMP) as intermediates occurred during the reaction. Their investigations demonstrated that TMP was formed from MMF under specific reaction conditions, which indicates that both TMP and MMF promote the synthesis of ML. When the reaction system was carried out using FAL and ethanol to prepare EL, the observation of the above-mentioned intermediates proposed in the mechanism was difficult. However, the published studies established two clear observations. The formation of the reaction intermediates (TEP and/or EMF) was slower than the reaction converting FAL into multiple intermediates. In addition, the yield of DEE formed in the reaction when compared to EL or the reaction intermediates (TEP and EMF) was low and various pathways contributed to the formation of EL.

**Figure 3:**
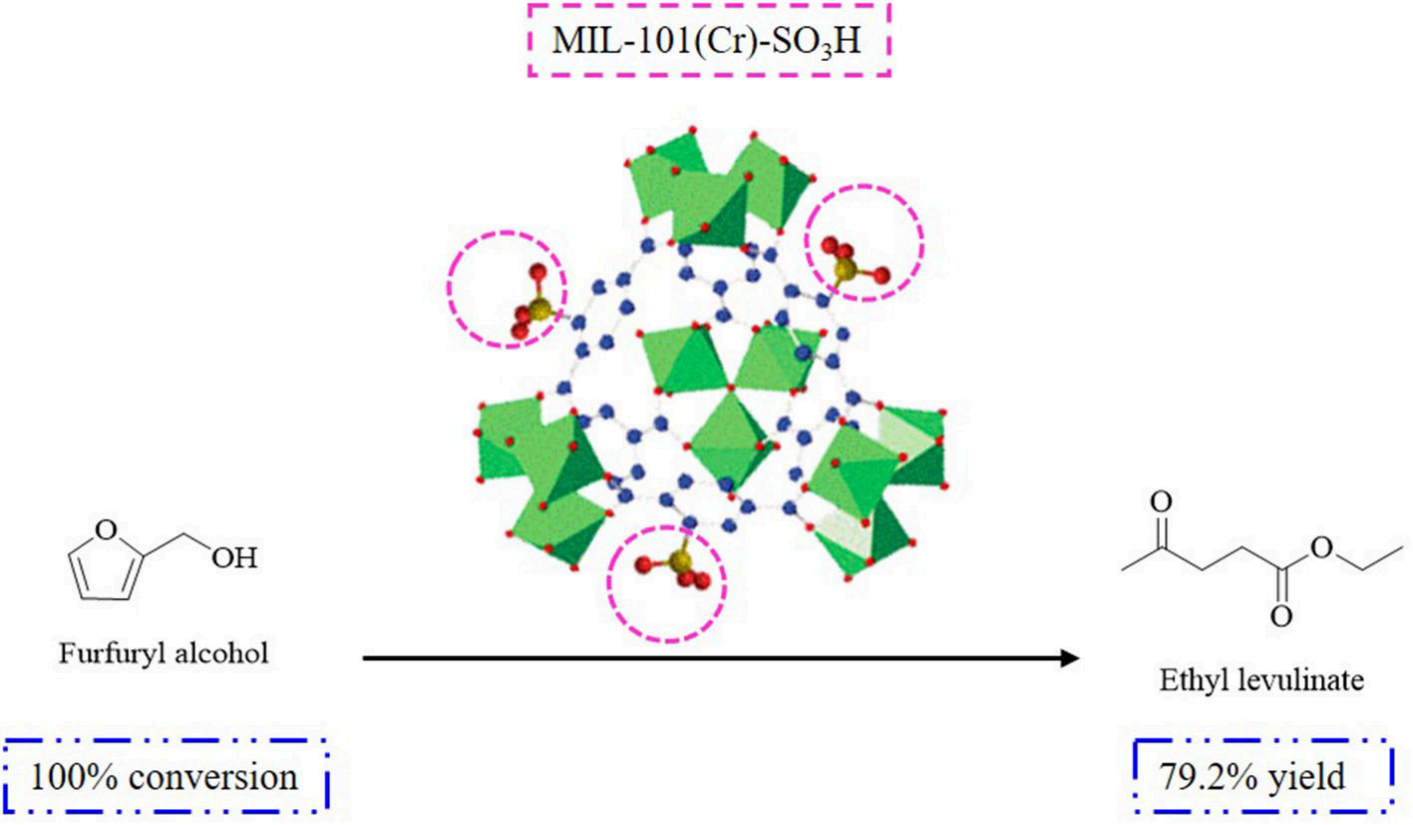
Reaction route for the conversion of FAL to EL over MIL-101(Cr)-SO_3_H.

The production of DEE during the synthesis of EL depends on the texture of the catalyst used, including the pore structure and accessibility of the active sites for ethanol dehydration derived from the ethanolysis of FAL. The results revealed the acid catalysts used for the FAL-EL procedure, reaction temperature, time, acidic sites employed, and observed EL yields. Owing to the microporous and non-swelling structure of gel-type resins (Dowex, Amberlyst, etc.), a higher amount of DEE was formed and a lower EL yield (<60%) was obtained, which can be ascribed to the improved accessibility of the active sites to ethanol when compared to FAL (Lange et al., 2009). This was different from resin catalysts with mesoporous structures (carbon-based, silica-based, etc.), which showed better EL yields (>80%) and lower amounts of DEE depending on the superior accessibility of the acidic sites to FAL when compared to ethanol (Lange et al., 2009; González Maldonado et al., 2012). Sulfonic acid-functionalized mesoporous materials (activated carbon, silica-carbon composites, organosilica hollow nanospheres, and sulfated MOF) have been used in the production of EL utilizing the ethanolysis procedure giving EL yields in the range of 80–90% (Russo et al., 2014a,b; Zhu et al., 2014; Liu et al., 2016; Guo et al., 2020). In addition, the density of the acidic groups has been shown to affect the reaction rate. For example, adjusting the –SO_3_H density from 5.4 to 17% in propylsulfonic acid-functionalized ethane bridged organosilica hollow nanospheres (Pr-SO_3_H-Et-HNS) gave EL yields ranging from 52.5 to 72% under the same reaction conditions. Meanwhile, with the catalyst MIL-101(Cr)-SO_3_H, EL yield was enhanced up to 79.1% when FAL was used as the starting material ascribed to the MOF texture (S_BET_ = 1,492 m^2^·g^−1^, titration [mmol(H+)·g^−1^] = 1.01) (Figure 3).

Sulfonic acid-functionalized ILs, which have similar non-swelling properties, have also been explored by Wang et al. to obtain better EL yields (Wang et al., 2014). A comparison of sulfonic acid and non-sulfonic acid-functionalized IL catalysts bearing ${\text{HSO}}_{4}^{-}$ anions was carried out. Using 3-butyl-1-methyl-1H-imidazole-3-ium [BMIm] as the cation gave a low EL yield (~34%) at 120°C, while replacing [BMIm] with BMIm-SH produces a significantly greater yield of EL (92%) under the same reaction conditions (Wang et al., 2014). The higher yield of EL obtained using the sulfonic acid functionalized IL was ascribed to the higher acid intensity. The acid intensities of BMIm-SH and BMIm IL were 1.2 and 2.5, respectively when measured using the Hammett acidity, which was comparable to conventional acids (i.e., H_2_SO_4_) (Liu et al., 2008). Subsequently, the results indicated that the cationic sulfonic group facilitates proton transfer and the protonation of FAL, which then enhances the yield of the target product (Li et al., 2015a,b). Similar tendencies were verified by Hengne et al. who showed that the EL yield obtained over a non-sulfonic IL (1- methyl imidazolium [MIm]) was lower (65%) than that obtained using a sulfonic acid-functionalized IL (95%) (Hengne et al., 2013).

Possessing both Brønsted and Lewis acidity, metal salts have been investigated in the acid-catalyzed transformation of biomass-derived oxygenates (Loerbroks et al., 2014). During the reaction, the Lewis acid active site coordinates with the oxygen atom in FAL, which was verified by the stability of the as-formed complex and further decreased the activation barrier (Pidko et al., 2010). To confirm the effect of the Lewis acidity, Peng et al. researched various aluminum salts (AlCl_3_, Al_2_(SO_4_)_3_, Al(NO_3_)_3_, and Al(C_6_H_5_SO_3_)_3_) as catalysts for the FAL-EL conversion reaction. A maximum EL yield of 74% was obtained using AlCl_3_ as a catalyst at 110°C for 3 h. This was attributed to the weaker Brønsted acidity of AlCl_3_ (pH = 0.89) compared to Al(OTf)_3_, which shows greater Brønsted acidity (pH = 0.12) (Peng et al., 2015). The EL yield obtained using AlCl_3_ was maintained over six successive runs of the reaction, demonstrating its high stability, which fulfills the requirements of its future commercialization (Peng et al., 2015). For comparison, Khusnutdinov et al. obtained a higher EL yield (95%) under milder reaction conditions (70°C) using iron(III) acetylacetone (Khusnutdinov et al., 2007).

Heterogeneous acidic catalysts, such as zeolites, have been recently studied for the ethanolysis of FAL used to produce EL. An excellent catalytic performance has been demonstrated using hierarchical HZ-5 and HZSM-5, which give EL yields up to 73.0 and 85.8%, respectively at 100–120°C (Zhu et al., 2014; Nandiwale et al., 2015). Nandiwale et al. were interested in the preparation of zeolite materials upon adjusting the Si/Al ratio (SAR) and further explored the effect of their Brønsted acidity on the EL yield (Nandiwale et al., 2015). A significant achievement was reported using the zeolite prepared with a SAR of 30.15 exhibiting the strongest Brønsted acidity (~0.73 mmol·g^−1^), which gave the maximum EL yield when compared to the other zeolites studied. Based on the published findings, the overall yield of EL obtained over the zeolite catalysts was affected by the formation of DEE, which leads to a serious loss of ethanol. These limitations can be overcome by modifying the textural properties of the zeolite materials. Antunes et al. have prepared and perfected a series of zeolite catalysts by introducing three different procedures to change the textural properties of the catalyst (Antunes et al., 2015, 2016). By introducing an organic template, Al-TUD-1 generated a smaller pore size distribution to increase the accessibility of the acidic sites to FAL, which produced a higher EL yield. Beta/TuD-1 was prepared with a reduced crystallite size and ITQ-2 was synthesized via the delamination of MCM-22 (Lima et al., 2010a,b; Antunes et al., 2012; Neves et al., 2013). Among the three catalysts studied, Al-TUD-1 exhibited a SAR of 21 and an acidity density of 197 μmol·g^−1^ and provided a significant yield of EL (80% over 24 h with negligible DEE formed) at 140°C (Neves et al., 2013). The delamination of MCM-22 was similar to Al-TUD-1, which opened the pore structure to provide increased accessibility to FAL (Corma et al., 1998). The surface area and the total acidity of the ITQ-2 catalyst were enhanced two- and 1.5-fold, respectively when compared to MCM-22. The results were in accordance with those reported by Katz et al. using layered zeolite and the delamination procedure was shown to be responsible for the enhancement in the acidity and surface area (Ogino et al., 2013). As a consequence, a significant enhancement in the EL yield (60%) was obtained using ITQ-2 when compared to MCM-22 (47%) at 140°C over 24 h (Neves et al., 2013).

Based on the above analysis, modified mesoporous aluminosilicates materials with high surface areas and appropriate pore sizes guaranteed the accessibility of the active sites for the reactants, which significantly improves the yield of EL. However, the production of undesired by-products containing aromatic compounds, cyclic hydrocarbons, and lactic acid poly-condensation compounds need to be inhibited to enhance the yield of EL (Dusselier et al., 2015).

Zhu et al. have also studied functionalized graphene oxide (GO) as an acid catalyst for the conversion of FAL into EL. The results showed that lamellar-structured nano-GO is beneficial for the accessibility of the reactants. Under the optimal reaction conditions (120°C), a high EL yield of up to 95.5% was obtained when compared to the other catalysts studied (modified zeolites, HPAs, and sulfonic acid functionalized catalysts; Zhu et al., 2014). The strong hydrogen bonding interactions formed by the synergistic effect observed between the various acid sites including sulfonic acid groups and carboxyl/hydroxyl functionalities are responsible for the excellent performance of the GO catalyst. The carboxyl/hydroxyl groups in GO have a strong affinity toward the hydroxyl group in FAL, which provides unprecedented adsorption and favors the accessibility of the active acid sites for the reactants (Zhu et al., 2015). In regard to the Brønsted acidity, the sulfonic acid density of GO is lower than H_2_SO_4_ and *p*-TSA, however, the higher yield of EL obtained using GO can be attributed to the crucial role played by this synergistic effect.

In addition to the catalytic performance, the recyclability of the catalyst is also a significant factor for its industrial applications. For instance, DH-ZSM-5 is reusable over five continuous runs without any significant loss in its activity. After the sixth run, the conversion of FAL decreased from 95 to 93%, demonstrating that the recycling of DH-ZSM-5 was feasible (Nandiwale et al., 2014). On the contrary, the sulfonated carbon catalyst (AC-Fe-SO_3_H) reported by Zhang and Chen, for which the yield of EL decreased from 58 to 46% after three runs, showed insufficient stability for industrial application (Zhang and Chen, 2016). Similarly, the EL yield decreased from 45 to 36% over six runs using USY zeolite (Chang et al., 2015). Further exploration is needed to achieve stable, convenient, and excellent catalytic performance during the conversion of FAL into EL.

#### CMF as a Feedstock

Besides the existing substrates, researchers have devoted great efforts toward developing the synthesis of EL from low-cost and novel starting materials, such as CMF (Li H. et al., 2014). For example, Breeden et al. have developed the synthesis of CMF (>70% yield) from HMF, glucose, and inulin at 80°C over 15 min (Breeden et al., 2013). Mascal et al. successfully obtained high EL yields up to 84.7% from CMF at 160°C over 30 min (Mascal and Nikitin, 2010a). The investigators confirmed the feasibility of a one-pot process to produce EL directly from biomass, even seed oil, using CMF as an intermediate (Mascal and Nikitin, 2010a). At room temperature, the obtained CMF intermediate remained stable in high yield (Mascal and Nikitin, 2010a), while at elevated temperature (>100°C), the ethanolysis reaction proceeds to convert the CMF intermediate into EL via TEP during the FAL ethanolysis process.

#### Saccharides as a Feedstock

With respect to the starting material, EL can be directly prepared from saccharides, such as carbohydrates, sucrose, glucose, or fructose *via* a one-pot process using an acid catalyst and alcohol as the reaction media. The one-pot process generates multiple by-products, which result in a low EL yield (Liu et al., 2011; Peng et al., 2011). Fructose has been reported to display excellent activity over multiple acid catalysts when compared to other monosaccharides (glucose and mannose).

Similar to the FAL conversion reaction, sulfonic acid functionalized ILs bearing [BMIm-SO_3_H] and [NE_t3_B-SO_3_H] cations have been developed to generate a comparable yield of EL ranging from 67 to 77% starting from fructose (Saravanamurugan et al., 2011). The difference observed in the EL yield was attributed to the acid intensity of the anions ([OMS]^−^, [HSO_4_]^−^, and [NTf_3_]^−^). Owing to the uniform structure of SBA-15 bearing sulfonic acid functionalities, its high Brønsted acid density (731 μmol·g^−1^) and surface area (819 m^2^·g^−1^) provide EL in 70% yield under the optimum reaction conditions (140°C over 24 h) (Saravanamurugan and Riisager, 2012). Similarly, Liu et al. have introduced a sulfonic acid functionalized Amberlyst-15 catalyst for the preparation of EL in ~73% yield (Liu et al., 2013). To explore the effect of the acid density on the formation of EL, a series of sulfonated mesoporous carbon catalysts were synthesized with decreasing acid site density (5.67, 4.26, 2.89, and 1.75 mmol·g^−1^). A declining trend in the EL yield (84, 69, 60, and 45%) was observed in the presence of the sulfonated mesoporous carbon structures (Liu et al., 2013).

The observed results indicate that the EL yield increases upon increasing the reaction time and temperature. For instance, the EL yield was improved to 37% when the reaction was conducted at a higher temperature (140°C) when compared to 130°C in the study reported by Zhu and coworkers (Wang et al., 2013). Furthermore, the utilization of a metal promoter on HPA can also afford a better EL yield. The results achieved by Zhao et al. showed that doping HPA with monovalent potassium cations (K^+^) leads to an increase in the acidity and higher EL yield (64.6%), which was further improved to 68.7% because of the use of toluene and heating at 150°C (Zhao et al., 2015).

Li et al. developed a fructose-EL conversion reaction catalyzed by HY zeolites with a SAR of 2.6 at 230°C, which achieved a 52% yield of EL (Li et al., 2016). The results were in contrast with the better EL yield (>80%) obtained at a lower temperature (<130°C) using LA and FAL as starting materials using similar zeolite-based catalysts. The reason for the lower yield can be attributed to the microporous structure of the Y-zeolite, in which the undesired 5-ethoxymethylfurfural (EMF) intermediate was formed and affected the rehydration step (Saravanamurugan and Riisager, 2012). Interestingly, H-USY zeolite has a superior BET surface area (732 m^2^·g^−1^) and acid density (1383 μmol·g^−1^), which exhibits better textural properties, to give a higher EL yield (~52%) at 160°C when compared to the weaker zeolite (699 m^2^·g^−1^, 874 μmol·g^−1^, EL yield ~40%) (Saravanamurugan and Riisager, 2012, 2013; Li et al., 2016). Thus, the structural characteristics including the pore structure, surface area, and acid site density are significant toward obtaining higher EL yields over zeolite materials, which indicates that the microporous structure of zeolite catalysts may suppress the complete conversion of fructose into the key intermediates of the reaction.

When compared with fructose, the alcoholysis of glucose to prepare ALs has been demonstrated to be difficult. The experimental results confirm that the EL yield can be significantly decreased (<13%) utilizing the same IL catalysts ([BMIm-SO_3_H] and [NEt_3_B-SO_3_H]) when the substrate was changed from fructose to glucose under identical reaction conditions (Saravanamurugan et al., 2011). The low EL yield derived from glucose was attributed to the formation of ethyl-D-glucopyranoside (EDGP) in the presence of a Brønsted acid (Li et al., 2015c). Similar to the isomerization of glucose in fructose, EDGP isomerization to ethyl-D-fructofuranoside (EDFF) is catalyzed by a Lewis acid (Morales et al., 2014). Generally, the isomerization of EDGP into EDFF is difficult and is considered to be a rate-limiting step (Li et al., 2015c). Therefore, the combination of both Brønsted and Lewis acid sites determines the successful conversion of glucose into EL (Zhao et al., 2015). Similar to the results obtained using ILs, several other catalysts have been investigated for the glucose alcoholysis reaction, which gave low yields of the target ALs. Interestingly, HPA acts as a catalyst over a shorter reaction time (120 min) to achieve an equal yield of EL (Yang et al., 2012; Wang et al., 2013), which was attributed to the existence of both Lewis and Brønsted acid sites and the appropriate Brønsted to Lewis acid site ratio (B/L >58) (Tao et al., 2015).

Zeolites possessing both moderate Brønsted and Lewis acidity have been explored by Xu et al. in the glucose ethanolysis reaction used toward the production of EL (Xu et al., 2013). An optimum EL yield of 40% was afforded at 180°C for over 30 min using USY zeolite starting from glucose. The moderate B/L ratio (~3.7) of the USY zeolite was responsible for the good EL yield (West et al., 2010; Otomo et al., 2015). When further mineral acid (H_2_SO_4_) was added to the USY zeolite, a higher EL yield of 51.4% was achieved over 120 min because of the increased B/L ratio (Chang et al., 2015).

The amount of Brønsted acid in the catalyst plays an important role during the ethanolysis of glucose and fructose, giving different EL yields. This has been demonstrated during the ethanolysis of disaccharides, such as sucrose. For instance, in the presence of sulfonic acid functionalized ILs, the EL yield achieved after 24 h via ethanolysis of sucrose was 43% at 140°C (Saravanamurugan and Riisager, 2012). This result was attributed to the molecular components in sucrose, which includes one molecule of glucose and one molecule of fructose. The EL yield (~43%) obtained from sucrose was higher than that from glucose (<13%) and lower than that from fructose (>70%). This indicates that the glucose molecule in sucrose produces EDGP, which is the rate-limiting step and difficult to isomerize, while the fructose molecule is easily converted into EDFF, which may undergo subsequent reactions to form EMF and EL. In accordance with this conclusion, Chen et al. published a similar EL synthesis (~45%) using Brønsted acidic IL-based HPAs [3.2H]_3_(PW_12_O_40_)_2_ (IL POM) to convert sucrose into EL (Chen et al., 2014). Generally, in the presence of only a Brønsted acid, the fructose molecule in sucrose can be converted into EL without the transformation of glucose. For example, sulfonated SBA-15 has been applied as a catalyst and the yield of EL from sucrose decreased (~35%) when compared to that obtained from fructose (Saravanamurugan and Riisager, 2012). However, in the presence of both Brønsted and Lewis acid sites, two molecules of sucrose can simultaneously undergo ethanolysis to form EL (Li et al., 2016). Different from sucrose, maltose contains two units of glucose, whose properties are similar to glucose. Therefore, the reactivity of maltose is similar to glucose, providing a better yield of EL when using the catalyst with an optimum ratio of Brønsted and Lewis acidity. The experimental exploration concluded by Hu et al. using maltose confirmed the above-mentioned hypothesis that a lower yield of EL (20%) is obtained in the presence of Amberlyst-70 when compared with the EL yield (47%) observed using H-USY (Hu et al., 2013; Saravanamurugan and Riisager, 2013).

Saravanamurugan et al. have studied polysaccharides constructed from fructose monomers, such as inulin, which provide excellent EL yields. The production of EL was found to conform to the same tendency as the Brønsted acidity of the catalyst used. For instance, high EL yields of up to 39.0, 52.3, and 67.0% have been obtained over H-USY zeolite, HPA, and IL functionalized polyoxometalate salts (IL-POM), respectively (Saravanamurugan and Riisager, 2013; Chen et al., 2014; Zhao et al., 2015). The Brønsted acidity followed the order of H-USY < HPA < IL-POM. Considering that cellulose is a glucose-based polysaccharide, the ethanolysis reaction was different under mild conditions for the production of EDGP and EL. Thus, the ethanolysis of cellulose conducted at temperatures >170°C can offer an appreciable EL yield. Deng et al. reported an EL yield of 27% via the ethanolysis of cellulose using HPA (H_4_SiW_12_O_40_) at 205°C over 30 min (Deng et al., 2011). However, multiple undesired by-products which formed via polymerization may affect the overall EL selectivity, which was responsible for the low EL yield gained during cellulose ethanolysis using metal-doped HPA as a catalyst at an elevated temperature of 220°C (Zhao et al., 2015). Consequently, Amarasekara et al. introduced ethanol and water as the solvent medium for the ethanolysis of cellulose using an IL (1-(1-propylsulfonic)-3-methylimidazolium chloride) as a catalyst. The existence of water favored the isomerization of glucose into fructose and avoided the formation of EDGP, resulting in a high yield of EL (38.5%) (Amarasekara and Wiredu, 2014).

Le Van Mao et al. have developed two different approaches for the one-pot conversion of lignocellulosic biomass into EL catalyzed by an acid catalyst (Le Van Mao et al., 2011). The first method showed that biomass can be directly converted into EL in ethanol under acidic catalysis, while the second method proposed that biomass was first hydrolyzed to produce a complex mixture, which then undergoes the ethanolysis reaction. Both approaches used woody biomass or grass and were conducted in a high-pressure batch reactor in the presence of sulfuric acid at 190°C for 2 h. The best yield was 16.6 wt.%.

Chang et al. have investigated the direct conversion of wheat straw in one-pot to produce EL using H_2_SO_4_ as a catalyst (Chang et al., 2012). Under the optimal reaction conditions, a maximum EL yield of 17.9 wt.% was observed from wheat straw. No significant increase in the EL yield was obtained upon increasing the sulfuric acid loading (Chang et al., 2015).

## Conclusions and Perspectives

This review summarizes the trends observed for the yields obtained for ALs from various starting materials including LA, FAL, CMF, monosaccharides, disaccharides, polysaccharides, and biomass residues using acid catalysts in the alcoholysis reaction. The esterification reaction used to produce EL mainly depends on the acid intensity, acid site density, and accessibility of the acid sites. The conversion of FAL depends on the intermediates obtained during the reaction. Glucose is difficult to react when compared to fructose, which avoids the isomerization step. An appropriate combination of Lewis and Brønsted acidity is needed for the transformation of glucose-based saccharides.

We can conclude that efficient catalytic procedures for the one-pot conversion of biomass directly into ALs and the separation and purification of ALs are urgently needed. Due to the complex structure of biomass and multifunctional active sites in the catalyst, green and sustainable process are highly desirable. As an alternative reaction approach, microwave irradiation has exhibited significant potential for reaction time reduction with fewer undesired products. The extensive exploration of microwave reactions for the transformation from lab scale to the industry scale to the synthesis of ALs needs to be studied further.

## Author Contributions

XL performed critical reviews and wrote the manuscript. CL and HW designed the structure of the manuscript and is responsible for the work. WY and QZ assisted XL in preparing and completing the review. All authors discussed the results, wrote, and commented on the manuscript.

## Conflict of Interest

The authors declare that the research was conducted in the absence of any commercial or financial relationships that could be construed as a potential conflict of interest.
